# Mismeasured Covariate in the Long-Term Survival of Colorectal Cancer

**DOI:** 10.22086/gmj.v8i0.1413

**Published:** 2019-07-08

**Authors:** Mehdi Azizmohammad Looha, Mohamad Amin Pourhoseingholi, Seyyed Vahid Hosseini, Soheila Khodakarim

**Affiliations:** ^1^Department of Biostatistics, School of Allied Medical Sciences, Shahid Beheshti University of Medical Sciences, Tehran, Iran; ^2^Gastroenterology and Liver Diseases Research Center, Research Institute for Gastroenterology and Liver Diseases, Shahid Beheshti University of Medical Sciences, Tehran, Iran; ^3^Department of Surgery, Gastroenterohepatology Research Center, School of Medicine, Shiraz University of Medical Sciences, Shiraz, Iran; ^4^Department of Epidemiology, School of Public Health and Safety, School of Allied Medical Sciences, Shahid Beheshti University of Medical Sciences, Tehran, Iran

**Keywords:** Colorectal Cancer, Errors, Survival Rate, Survival Analysis

## Abstract

**Background:**

Colorectal cancer (CRC) is one of the most important causes of morbidity and mortality worldwide. This study aimed to determine the effect of measurement error of risk factors on the cure fraction of CRC patients.

**Materials and Methods:**

This study was conducted using the medical records of 346 patients with CRC, who were followed up between 2006 and 2017 in Shiraz, Iran. In our data, lymph node ratio (LNR) was a characteristic measuring with error. This variable was used in the model with 0.04 and 0.8 of error variance. Nonmixture nonparametric cure rate model and its corrected forms, simulation-extrapolation (SIMEX) and corrected score (CS), were applied to the data.

**Results:**

In noncured cases, the mean survival time was 1115.45 (95% confidence interval, 1043.60-1187.30) days. The 1-, 3-, and 5-year survival rates were 0.93, 0.71, and 0.65, respectively. The proportion of cured patients was 65.2%. The SIMEX method did not change the effect of LNR substantially on cure fraction as compared with the naive method when the variance of measurement error was 0.04 and 0.80. The CS method changed the effect of LNR on cure fraction even when the variance of measurement error was 0.04.

**Conclusion:**

The best method to assess the effect of LNR on cure fraction was the naive method, and the CS method was not deemed to be a valid method to correct the measurement error in LNR.

## Introduction

Colorectal cancer (CRC) is considered as the third and second most commonly diagnosed malignancy in men and women, respectively. Likewise, it is the second leading cause of mortality related to cancers, with 1.8 million new cases and 881,000 deaths estimated to have taken place in 2018 [1]. The CRC incidence rate fluctuates at different times and across populations worldwide. Also, it ranges from more than 40 per 100,000 persons in the United States, Western Europe, and Australia to less than 5 per 100,000 persons in Africa and some parts of Asia [2]. The incidence rates in different populations may change over time [3]. Geographically, the global distribution of CRC is different. CRC has been found to be prevalent in developing countries influenced by western culture [4]. However, the developed countries account for the majority of CRC cases [1]. Typically, the CRC survival is fully dependent on its stage at diagnosis. In general, early diagnosis can increase the chance of survival in patients with CRC [5]. Epidemiology studies have indicated that inflammatory bowel disease, history of CRC in first degree relatives, obesity, physical activity, smoking, heavy alcohol use, and age over 50 years are the potential risk factors of CRC [6, 7]. The CRC is the third and fourth most common cancer in Iranian men and women, respectively. According to the Iranian National Cancer Registry report, the incidence of CRC has markedly increased during the past 25 years. [8-10]. In addition, it has increased from 813 cases in 2000 to 6120 cases in 2009 [11]. The statistics clearly indicate that the incidence of CRC is on the rise in Iran; however, it varies from region to region [12]. Although CRC usually occurs after the age of 55 [13], it is estimated that half of the patients with CRC in Iran are younger than 50 years [10]. A decrease in CRC mortality rate has been reported in many countries, and some patients have been cured [2, 14]. In the United States, the 5-year and 10-year survival rates for CRC patients were 65% and 58%, respectively. However, survival rates for CRC can vary based on a variety of risk factors [15]. The 5-year survival remained at approximately 60% in the last decade in Asia [16]. Therefore, survival analysis is a proper tool to identify the potential risk factors of this fatal disease. Most of the researchers often work with survival data, which measure the time until the event of interest occurs. In classical survival analysis, if the follow-up time is sufficiently long, all subjects will eventually experience the event of interest; therefore, the population survival function will tend toward zero at infinity, whereas other common status in the analysis of time-to-event data occurs when subjects would never experience the event of interest. Usually, these patients are termed as long-term survivors, immune, or cured. For such data, the survival curve has a stable plateau at the end of the study; therefore, we cannot use the classical survival model. In this case, the cure models are used instead of classical survival models, which consider the presence of cured subjects in the population of interest [17]. The estimation of the cure fraction in cancer research is very important in providing information to patients and monitoring survival trend over time [18]. Despite the fact that most of the research in medical science focuses on the investigation of the effects of the prognostic factors on the outcome, some of these factors usually are measured with error. These errors, called measurement errors, can be caused by using imprecise devices or as a result of using imprecise methods for measuring the quantity of interest. The lymph node ratio (LNR) as a prognostic factor for CRC is known to be measured with error. If we use the covariates with measurement error in a statistical model, it leads to several consequences [19], including bias in the estimation of the covariates effect. This may contribute to the fact that a covariate with a significant effect is considered nonsignificant [20]. In this study, we have estimated the effect of the LNR in promotion time cure model and compared it with the estimated effect in corrected-versions on patients with CRC.

## Materials and Methods

All patients with colon and rectal malignancies referred to the Shahid Faghihi Hospital in Shiraz from January 2006 had been registered in the Colorectal Research Center of Shiraz. Their information contained demographic variables and history of the disease along with clinical symptoms and clinical history of diagnosis, age at diagnosis, surgical information, as well as pathologic data such as the appearance of the tumor, tumor size, number of isolated lymph nodes and number of lymph nodes involved, the size and stage of the tumor, and lymphatic and vascular invasion of the tumor. Each patient was visited in the first year after surgery once every 3 months and in the second year every 6 months; thereafter, their information was recorded by the colorectal surgeon annually. In the case of no referral, the research center staff contacted the patient or patient’s family at certain intervals by telephone and informed them about the patient’s current condition. If the patient was dead, the date was recorded, and if he or she was referred to other treatment centers for follow-up, the necessary medical records and information would be collected and then registered after physician’s confirmation. The data of this retrospective study are for 346 patients diagnosed with CRC from January 2006 and followed up until February 2017. Deaths due to CRC were regarded as failure, and survival time was calculated as the time interval between CRC surgery and death due to CRC. The stepwise selection was implemented to determine the best subset of variables in which the best proportional hazard model was obtained. The variance inflammatory factor (VIF) was used to evaluate the presence of multicollinearity between the variables. Variables affected by multicollinearity were removed from the study. This study approved by the Ethics Committee of the Shahid Beheshti University of Medical Sciences (approval code: IR.SBMU.PHNS.REC.1396.91). There are 2 cure model approaches, which are useful tools to analyze and describe cancer survival data. The first one is the standard cure rate model or mixture cure model which divides the population into the cured and non-cured group [21]. The second one is promotion time cure model or nonmixture cure model [22]. In this study, we focused on promotion time cure model and 2 corrected promotion time cure approaches, corrected score (CS) and simulation-extrapolation (SIMEX). They are used when 1 (or more) covariate(s) are measured with error. The backfitting algorithm for maximizing likelihood is used in these semiparametric approaches [23]. In measuring patients’ characteristics, usually, there is no information about the distribution and variance of measurement error in the LNR; however, in SIMEX approach, we can use normal distribution for measurement error, which is robust to other distributions [19]. The SIMEX algorithm has several advantages, which makes it a very appealing method compared with other cure models. However, the CS method should be used, based on some assumptions. SIMEX yields the lowest mean square error (MSE) when the variance of measurement error is relatively large. In the smaller values of variance, the naive method, which does not consider the measurement error, yields the lowest MSE. Therefore, when the measurement error variance is small, it is better not to perform any correction in terms of the MSE. In the small values of measurement error variance, the CS method yields the lowest bias. Consequently, the conclusions depend on the estimation method, the criterion (MSE or bias), the variance of measurement error, and on the covariates to be interested [24, 25]. The log(-log) link function was used in all 3 models; thus, the negative regression coefficients lead to a larger cure fraction, and the positive regression coefficients lead to a smaller cure fraction. LNR, the ratio of total involved lymph nodes, may be measured by error, but there is no information about the distribution and variance of this measurement error [23, 26]. Therefore, the variance of measurement error in LNR was considered 0.04 and 0.9. We aimed to investigate the promotion time cure model and its corrected versions in the dataset to detect this probable effect. Both correction methods were implemented in R packages miCoPTCM version 1.0 (https://cran.r-project.org/). We described the categorical and numerical characteristics with frequency (percentage) and mean (SD), respectively. The significance level was set at P≤0.05.

## Results

**Figure 1:**
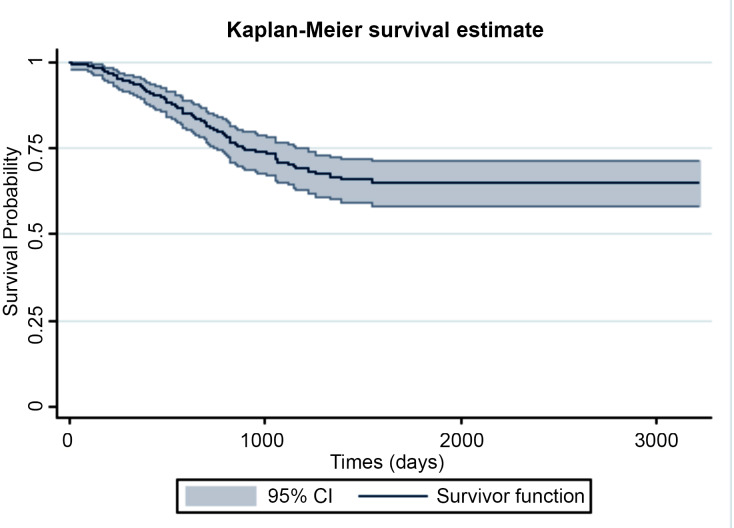
Kaplan-Meier survival curve of the entire study population

**Figure 2:**
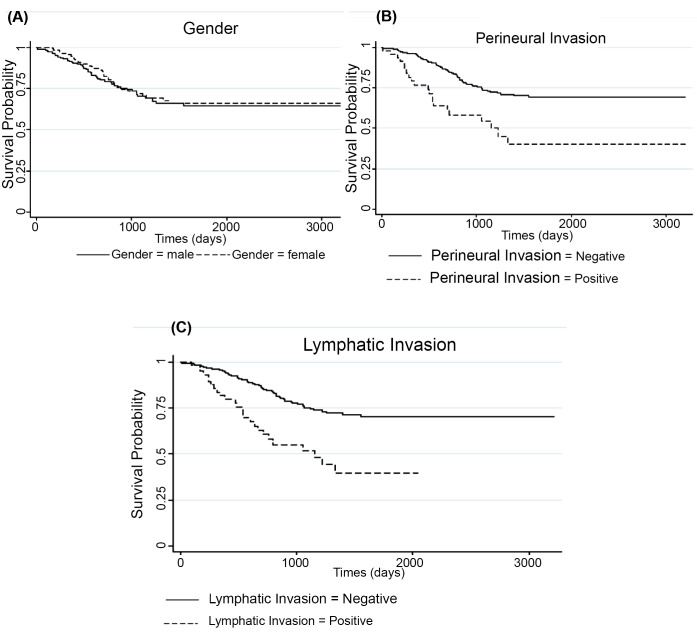
Survival probability curve by gender (**A**) by perineural invasion (**B**) by lymphatic invasion (**C**).

A total of 346 patients with CRC were enrolled in the study. The mean age of patients was 57.40 (95% confidence interval [CI], 56.0-58.8), and the proportion of men (55.78%) was higher than women (44.22%). For uncured patients, the mean and median survival time were 1115.45 days (95% CI, 1043.60-1187.30) and 1258.00 days (95% CI, 1192.37-1324.63), respectively. The 1-, 3-, and 5-year survival rates were 0.93 (95% CI, 0.90-0.96), 0.71 (95% CI, 0.66-0.77), and 0.65 (95% CI, 0.59-0.72), respectively. Age at diagnosis, hemoglobin (Hb), body mass index (BMI), LNR, perineural invasion (PNI), lymphatic invasion, and gender were selected as risk factors after applying stepwise selection and VIF analysis. Table-1 indicates the results of descriptive statistics for categorical and numerical variables. A plateau in the Kaplan-Meier survival curve begins at approximately 4 years, which confirms the presence of cured patients (Figure-1). It means that cure rate models led to more accurate results than classical survival models. In addition, the longest follow-up time was 3224 days. Besides, this figure shows that the overall observed “cured” proportion of the patients with CRC was 65.2% (95% CI, 59.1-72.0). Based on the existing references, the lymph node may have been measured by error; therefore, the naive method, which does not take any measurement error into account, and the CS and SIMEX methods, which take measurement error into account, were the 3 approaches we used to estimate cure fraction for patients with CRC. Furthermore, the variance of measurement error in the lymph node was considered 0.04 and 0.9 to detect this probable effect. Table-2 shows promotion time cure rate models: Naive, C, and SIMEX methods with 0.9 and 0.04 variance of measurement error in the LNR. When the variance of measurement error in the lymph node is 0.9, LNR, BMI, Hb, and PNI are significant, and the sign of effects on survival time are the same in naive and SIMEX methods. In the CS method, LNR, BMI, Hb, and lymphatic invasion are significant, and the coefficient of LNR is −0.117 (SE 0.036). The same result is observed in Table-3 when the variance of measurement error in the lymph node is 0.04. The coefficients of LNR and gender in naive and SIMEX methods are positive, but they are negative in the CS method. The effect of PNI variable on the survival of patients in the CS method is not significant, but it is significant in the naive and SIMEX methods. In Figure-2A, the survival rate is similar for males and females, but the probability of cured individuals is slightly higher in males than females. In addition, the coefficient of gender in naive and SIMEX methods in Table-2 is positive, showing higher cure probability in females compared with males, but in CS method, the coefficient of gender is negative, indicating a smaller cure probability in females compared with males. The latter result was in contrast with the results obtained from the curve by gender group. Figure-2B shows that the overall cured proportion for the negative PNI was 29.1% lower in comparison with the positive group (P<0.001). We found no association between the “cured” proportion and PNI in the CS method (P=0.056). However, there was a significant association in naive and SIMEX methods. Figure-2C shows that the cured proportion in positive lymphatic invasion was 39.8%, whereas it was 70.5% in the negative lymphatic invasion, and the difference between 2 survival curves was significant (P<0.001). Moreover, we found no association between the “cured” proportion and lymphatic invasion in the naive and SIMEX methods; however, the CS method showed a significant association between the cured proportion and the lymphatic invasion. The estimated cure fraction in the naive method was 71.5%; when the measurement error in LNR was 0.9, the estimated cure fractions in CS and SIMEX methods were 70.62% and 71.47%, respectively, and when the measurement error in LNR was 0.04, the estimated cure fractions in CS and SIMEX methods were 67.5% and 71.34%, respectively.

## Discussion

In this study, it appears that the conclusions do not change in naive and SIMEX methods, entirely. However, there are many differences between the results of the naive method and the CS method, especially when the measurement error variance is low (0.04). The CS method is not valid as this method yields unacceptable point estimates. Typically, when the variance of measurement error is low, we expect a very close estimate to the one that is obtained by the naive method. On the other hand, unlike the CS method, the SIMEX method does not change the sign of estimated gender variable. Therefore, the SIMEX method yields results that are more acceptable.Based on the results of SIMEX, the measurement error does not have any significant effect on the results of the naive method. It is not essential to consider the measurement error in the LNR. However, if we use the CS method, it causes biased decisions. In medicine, there are features in time-to-event data that would not let us apply the classical statistical tools of survival analysis. A total of 2 characteristics have been studied in this paper: the first one is the presence of the cured subject in the data, subjects who will never experience the event of interest, and the other characteristic is the presence of measurement error in 1 (or more) of the continuous covariates. The promotion time cure model has been used, which takes the presence of cured subjects into account. A total of 2 methods have been considered to estimate the parameters of the promotion time cure model with measurement error covariate. Our results show that the SIMEX method is the best correction, and CS method is not valid. In this study, the main objective is to investigate the impact of LNR variable on the survival of CRC when this variable is measured with error. The lymph node factor is of known importance in earlier cancer stages and is a significant marker for the survival prognosis. Also, the LNR is significant in stages 3 and 4 of the disease [27, 28]. Therefore, studying the effect of the LNR with the measurement error is very important. In our data, based on the SIMEX method (or naive), the LNR has the largest estimated positive coefficient, and this means that increasing this ratio reduces cure probabilities of patients. One of the risk factors for mortality of CRC is BMI (measured in kg/m2). Indeed, the increase in BMI is associated with increased risk for the incidence of CRC; BMI has been inconsistently associated with survival after CRC diagnosis. In a cohort study in the United States about the impact of BMI on survival after diagnosis of CRC, prediagnosis BMI was an important predictor of survival among patients with nonmetastatic CRC. However, postdiagnosis BMI was not associated with CRC mortality [29]. In a retrospective study in Iran, the results indicated that the BMI had a statistically significant effect on survival time, and decreasing BMI would increase the risk of patient’s death from CRC [30]. In this study, the BMI has a meager significant impact on cure rate of patients with CRC; thus, an increase in BMI leads to a slight increase in cure probability of patients. Obese patients may have better nutritional resources to withstand the devastating effect of cancer itself. Similar results obtained from a study implied that high BMI was associated with high median overall survival [31]. Typically, Hb changes after diagnosis (both decreased and increased Hb levels) had an adverse effect on patient survival [32]. A study conducted in the United States demonstrates that postdiagnosis Hb change is associated with the lower survival of CRC. In this study, increased Hb levels were associated with a slight increase in the cure rate of patients, but there is no clear explanation for this. It may be due to the fact that for a high percentage (over 80%) of male and females, Hb levels are below their normal range. PNI is another factor that usually has a significant effect on CRC survival time, which is associated with decreased survival in CRC, and patients with PNI-positive tumors experience lower survival [33]. Our data suggest that only 13% of patients had PNI-positive tumors, and the cure probability of death in PNI-negative tumors was less than cure probability of death in PNI-positive tumors.Moreover, although PNI-negative patients experience better survival than PNI-positive patients, it takes a longer time for them to reach the cure state. Lymphatic invasion can be used for evaluating tumor aggressiveness and estimating patient survival; it is clearly correlated with the disease stage [34]. In this study, 17% of patients had lymphatic invasion, and it has no significant impact on survival of patients with CRC after surgery. Among patients with CRC, increasing age is not independently associated with complications after surgery; however, in many studies, age was a significant predictor for the cause of death [35]. However, in the ongoing study, age had no significant effect on survival of patients with CRC. The mean age for patients who were dead and were cured was 58.8 and 54.3 years, respectively. It may be because of the fact that advanced stages of CRC have been observed at any age, and younger patients did not necessarily have an earlier stage of cancer. The proportion of cured patients and median survival time in our study was slightly higher than the study recently conducted in Iran [36].

There are not many issues about measurement error in the cure rate models. It has been studied in other fields, for example, the joint model of survival and longitudinal data with errors [37], the measurement error on risk prediction [38], etc. The limitation of this study is that some variables, such as tumor size and cancer stage were not properly collected; therefore, we could not use them in our study. Furthermore, because of the problem of multicollinearity, we could not use all variables, simultaneously. It is recommended to conduct further studies about the impact of mismeasured covariates in parametric cure model.

## Conclusion

Some characteristics associated with CRC may be measured with error. This error can cause bias because of the effect of mismeasured characteristics on cure fraction. In this study, LNR was known as a mismeasured characteristic, but in our data, this error in measuring did not have much effect on cure fraction.

## Acknowledgment

This study was supported by the School of Public Health and Safety, Shahid Beheshti University of Medical Sciences, Tehran, Iran (grant number=12534). Also, we would like to acknowledge the staff of Cancer Research Center of Shiraz University of Medical Science for their wholehearted cooperation during the data gathering.

## Conflict of Interest

None declared.

**Table 1:** Descriptive Statistics for Categorical and Numerical Variables

**Variables**	**Total of patients**	**Patients who died**
**Lymph node ratio**	0.124 ± 0.25^**^	0.232 ± 0.35
**BMI**	24.31 ± 5.31	22.58 ± 4.14
**Hemoglobin**	12.04 ± 2.08	11.47 ± 1.94
**Age at diagnosis**	57.40 ± 13.36	58.80 ± 13.93
**Perineural invasion**		
Negative^*^	299 (86.42)^***^	62 (74.7)
Positive	47 (13.58)	21 (25.3)
**Lymphatic invasion**		
Negative^*^	285 (82.37)	57 (68.7)
Positive	61 (17.63)	26 (31.3)
**Gender**		
Male^*^	193 (55.78)	48 (57.8)
Female	153 (44.22)	35 (42.2)

*Reference category, **Mean ± standard deviation, ***Number of patients (percent)

**Table 2:** Estimation Based on Naive, CS and SIMEX When Variance of Measurement Error in Lymph Node Ratio Is 0.9

	**Naive**			**CS**			**SIMEX**		
**Factors**	**estimate**	**S.E**	**P-value**	**estimate**	**S.E**	**P-value**	**estimate**	**S.E**	**P-value**
**Lymph node ratio**	1.529	0.341	<0.001*	-0.117	0.036	0.001*	1.530	0.345	<0.001*
**BMI**	-0.072	0.025	0.004*	-0.062	0.025	0.013*	-0.069	0.024	0.005*
**HB**	-0.138	0.056	0.014*	-0.148	0.058	0.011*	-0.174	0.064	0.007*
**Age**	0.012	0.008	0.121	0.014	0.008	0.100	0.012	0.008	0.149
**Perineural invasion**									
Negati^ve^**									
Positive	0.661	0.282	0.019*	0.557	0.292	0.056	0.645	0.285	0.024*
**Lymphatic invasion**									
Negative^**^									
Positive	0.539	0.288	0.061	0.973	0.270	<0.001*	0.533	0.292	0.068
**Gender**									
Male^**^									
Female	0.057	0.224	0.799	-0.080	0.230	0.727	0.044	0.224	0.845

**BMI:** Body mass index; **S.E:** Standard error *Significance at the 5% level, **Reference category

**Table 3:** Estimation Based on Naive, CS and SIMEX When Variance of Measurement Error in Lymph Node Ratio Is 0.04

	**Naive**			**CS**			**SIMEX**		
**Factors**	**estimate**	**S.E**	**P-value**	**Estimate**	**S.E**	**P-value**	**Estimate**	**S.E**	**P-value**
**Lymph node ratio**	1.529	0.341	<0.001*	-4.981	1.255	<0.001*	1.529	0.346	<0.001*
**BMI**	-0.072	0.025	0.004*	-0.058	0.029	0.048*	-0.069	0.025	0.005*
**HB**	-0.138	0.056	0.014*	-0.169	0.068	0.012*	-.179	0.068	0.008*
**Age**	0.012	0.008	0.121	0.015	0.009	0.100	0.011	0.008	0.163
**Perineural invasio**n									
Negative^**^									
Positive	0.661	0.282	0.019*	0.511	0.335	0.127	0.659	0.286	0.021*
**Lymphatic invasio**n									
Negative^**^									
Positive	0.539	0.288	0.061	1.524	0.367	<0.001*	0.542	0.293	0.064
**Gender**									
Male^**^									
Female	0.057	0.224	0.799	-0.286	0.269	0.288	0.038	0.225	0.866

**BMI:** Body mass index; **S.E:** Standard error *Significance at the 5% level, **Reference category
